# A Nod to disease vectors: mitigation of pathogen sensing by arthropod saliva

**DOI:** 10.3389/fmicb.2013.00308

**Published:** 2013-10-21

**Authors:** Olivia S. Sakhon, Maiara S. Severo, Michail Kotsyfakis, Joao H. F. Pedra

**Affiliations:** ^1^Department of Microbiology and Immunology, University of Maryland School of MedicineBaltimore, MD, USA; ^2^Department of Entomology, Institute for Integrative Genome Biology, Center for Disease Vector Research, University of CaliforniaRiverside, CA, USA; ^3^Institute of Parasitology, Biology Centre, Academy of Sciences of the Czech RepublicCeske Budejovice, Czech Republic

**Keywords:** Nod-like receptors, inflammasome, vector-borne pathogens, vector-borne diseases, arthropod saliva, salivary proteins

## Abstract

Arthropod saliva possesses anti-hemostatic, anesthetic, and anti-inflammatory properties that facilitate feeding and, inadvertently, dissemination of pathogens. Vector-borne diseases caused by these pathogens affect millions of people each year. Many studies address the impact of arthropod salivary proteins on various immunological components. However, whether and how arthropod saliva counters Nod-like (NLR) sensing remains elusive. NLRs are innate immune pattern recognition molecules involved in detecting microbial molecules and danger signals. Nod1/2 signaling results in activation of the nuclear factor-κB and the mitogen-activated protein kinase pathways. Caspase-1 NLRs regulate the inflammasome~– a protein scaffold that governs the maturation of interleukin (IL)-1β and IL-18. Recently, several vector-borne pathogens have been shown to induce NLR activation in immune cells. Here, we provide a brief overview of NLR signaling and discuss clinically relevant vector-borne pathogens recognized by NLR pathways. We also elaborate on possible anti-inflammatory effects of arthropod saliva on NLR signaling and microbial pathogenesis for the purpose of exchanging research perspectives.

## INTRODUCTION

Vector-borne diseases impact individuals worldwide and, with their frequencies increasing, they are becoming a crucial public health problem in need of attention (McGraw and O’Neill, 2013). With more than 200 million affected individuals, malaria is spreading rampant in tropical and subtropical regions and dengue fever is following close behind (**Table 1**). The spread of these illnesses, as well as other vector-borne diseases, has been attributed to rapid globalization, anthropomorphic and environmental changes, and the lack of effective vaccines (Kovats et al., 2001). These maladies have been combated by preventive care and therapeutics (Mejia et al., 2006; Fontaine et al., 2011). In order to develop novel treatments, scientists are continuously attempting to elucidate the mechanism of microbial transmission and aspects of the immune system that are involved in pathogen recognition (Titus and Ribeiro, 1990). Considering the variability between pathogens being transmitted from an arthropod vector to the mammalian host, one can imagine why the development of a vaccine has been an arduous task. However, vaccine development has taken a new route towards a common factor that all disease-transmitting vectors share: saliva. To promote feeding, hematophagous arthropods rely on salivary proteins to not only impart anti-hemostatic capabilities but also anti-inflammatory activities (Ribeiro and Francischetti, 2003; Chmelar et al., 2012).

**Table 1:** Nod-like receptors and vector-borne diseases of health and economic importance.

Disease	Pathogen	Vector	Number of affected individuals	Mortality	Nod-like receptor	Reference
1. Malaria	*Plasmodium* spp.	*Anopheles gambiae*	216 million	655,000	NLRP3 Nod1 Nod2	Ockenhouse et al. (2006), Coban et al. (2007), Dostert et al. (2009), Finney et al. (2009), Griffith et al. (2009), Shio et al. (2009), World Health Organization (2013a)
2. Dengue fever	Dengue virus	*Aedes aegypti, Aedes albopictus*	50 million annually	22,000	NLRP3	World Health Organization (2013b), Centers for Disease Control and Prevention (2012a), Wu et al. (2013)
3. West Nile neuroinvasive disease	West Nile virus	*Culex quinquefasciatus*	*	*	NLRP3	Demento et al. (2009), World Health Organization (2013c), Kaushik et al. (2012), Ramos et al. (2012), Centers for Disease Control and Prevention (2013c)
4. Leishmaniasis	*Leishmania* spp.	*Lutzomyia longipalpis*, *Phlebotomus papatasi*	12–15 million	60,000	NLRP3 NLRC4?	Centers for Disease Control and Prevention (2013b), Lima-Junior et al. (2013), Sani et al. (2013)
5. Chagas disease	*Trypanosoma cruzi*	*Rhodnius prolixus*	10 million	>10,000	Nod1	Silva et al. (2010), Aoki et al. (2012), Centers for Disease Control and Prevention (2010), World Health Organization (2013d)
6. *Lyme Borreliosis*	*Borrelia burgdorferi*	*Ixodes *spp.**	110,000**	1**	Nod2	Lindgren and Jaenson (2006), Cruz et al. (2008), Wilmanski et al. (2008), Liu et al. (2009), Berende et al. (2010), Oosting et al. (2011), Petnicki-Ocwieja et al. (2011), Centers for Disease Control and Prevention (2012b), The New York Times (2013)
7. Plague	*Yersinia pestis*	*Xenopsylla cheopis*	1,000–3,000 annually	80–300	NLRP12 NLRP3 NLRC4 Nod2	Ferwerda et al. (2009), Brodsky et al. (2010), Zheng et al. (2011), Centers for Disease Control and Prevention (2012c), Vladimer et al. (2012), Healthline (2013)
8. Human granulocytic anaplasmosis	*Anaplasma phagocytophilum*	*Ixodes *spp.**	1,000 annually***	<10***	NLRC4	Pedra et al. (2007), Centers for Disease Control and Prevention (2013d)
9. Tularemia	*Francisella tularensis*	*Dermacentor *spp., *Amblyomma americanum*	120*** 500,000	1–29***	AIM2 NLRP3	Fernandes-Alnemri et al. (2010), Atianand et al. (2011), Centers for Disease Control and Prevention (2011b), Center for Infectious Disease Research and Policy (2013), MD Guidelines (2013), Medscape (2013)
10. Yellow fever	Yellow fever virus	*Aedes aegypti*	200,000	30,000	NLRP1? NLRP3?	Gaucher et al. (2008), Centers for Disease Control and Prevention (2011a), World Health Organization (2013e)
11. Lymphatic filariasis	*Wuchereria bancrofti Brugia* spp.	*Culex *spp*., Anopheles *spp*., Aedes *spp*., Mansonia *spp.	120 million	—	Nod1 Nod2	Babu et al. (2009), Centers for Disease Control and Prevention (2013a)

*Estimates are not available.

**Estimates in the United States and Europe.

***Estimates in the United States.

*—Not applicable.*

*? Potential association, needs further confirmation.*

The relationship between arthropod saliva and components of the vertebrate immune system, such as Toll-like receptors (TLRs), has been studied. However, one crucial element of innate immunity that still remains vague, with regards to vector-borne diseases, is Nod-like receptors (NLRs). NLRs are an evolutionarily conserved mechanism for pathogen recognition found in both plants and mammals (Nürnberger et al., 2004). Since their discovery, numerous groups have identified the role of NLRs in the recognition of self-derived danger associated molecular patterns (DAMPs), such as ATP, and pathogen associated molecular patterns (PAMPs), such as those from fungi, bacteria, and viruses (Strowig et al., 2012). However, the association between NLRs and vector-borne pathogens still remains unclear. Only recently have researchers drawn attention to the detection of these pathogens by NLRs; even more ambiguous is the connection between salivary proteins and NLRs.

Here, we will address what occurs once a crucial barrier, the skin, is breached by an arthropod vector. We will discuss the subsequent recognition of key vector-borne pathogens by NLRs, and potential mechanisms by which salivary proteins may modulate this interaction. Though not all-encompassing, our focus is on acknowledging major examples by which saliva can modify immunity during infection. For a more comprehensive discussion about proteinaceous and non-proteinaceous salivary molecules, and their function during arthropod feeding, the reader is referred to accompanying reviews in this thematic research topic.

## ARTHROPOD SALIVA AND SALIVARY PROTEINS

Hematophagous arthropods have developed ways to promote the extraction of blood from their hosts while evading detection. The penetration of an arthropod mouthpart into the mammalian host promotes the release of saliva and allows for the acquisition of a blood meal. Though some components of saliva are ubiquitous to all arthropods, specific molecules for different vectors have also been reported (Mans and Francischetti, 2011). For over a hundred years, researchers have identified and dissected the components of saliva and found it to contain anti-hemostatic and anti-inflammatory properties (Sabbatani, 1899). In order to maintain a fluid supply of blood, salivary proteins act as vasodilators, inhibitors of platelet activity, and anti-coagulants (Champagne, 2005). To avoid recognition by the host, saliva not only modulates the inflammatory response, but it can also inhibit immune signaling (Chmelar et al., 2012). Arthropod saliva is composed of a plethora of salivary proteins that possess unique immunomodulatory functions (**Table 2**). Effects of tick saliva can been seen in a range of immune cell types, such as macrophages, neutrophils, T cells, B cells, and others (Gillespie et al., 2000; Titus et al., 2006; Chen et al., 2012). Salivary proteins with immunomodulatory properties from a myriad of arthropods, include but are not limited to: *Rhodnius prolixus*, *Rhipicephalus appendiculatus*, *Lutzomyia longipalpis*, *Aedes aegypti*, and *Anopheles gambiae *have been described. These proteins do not simply target one immune constituent but rather they span the gamut of cellular and molecular immunity.

**Table 2:** Salivary protein components and immunity.

Protein component	Vector	Cellular	Molecular	Reference
ISL929 ISL1373	*Ixodes scapularis*	Neutrophils	↓ Superoxide production ↓ β2-integrins	Guo et al. (2009)
ISAC	*I. scapularis*	Complement	Dissociates C3 convertase	Valenzuela et al. (2000), Soares et al. (2005)
IL-2 binding protein	*I. scapularis*	T cells	Binds IL-2	Gillespie et al. (2001)
Salp 25D	*I. scapularis*		Catalyzes the reduction of hydrogen peroxide with glutathione and glutathione reductase (antioxidant)	Das et al. (2001)
Salp20	*I. scapularis*	Complement	Dissociates C3 convertase	Tyson et al. (2007), Tyson et al. (2008)
Sialostatin L	*I. scapularis*	Neutrophils, dendritic cells, mast cells	↓ Neutrophil influx, CD80/86, IL-12p70, TNF-α, MHC II, cathepsin L, IFN-γ, IL-17, T cell proliferation	Kotsyfakis et al. (2006), Kotsyfakis et al. (2007), Sá-Nunes et al. (2009), Horka et al. (2012)
IsSMase	*I. scapularis*	T cells	↑ IL-4	Alarcon-Chaidez et al. (2009)
PGE_2_	*I. scapularis*	Dendritic cells	↓ IL-12, TNF-α, CD40, inhibitor of differentiation Induces cAMP-PKA signaling	Sá-Nunes et al. (2007), Oliveira et al. (2011)
Histamine release factor (HRF)	*I. scapularis Dermacentor variabilis*	Basophils, mast cells	Release of histamine	Dai et al. (2010), Mulenga et al. (2003)
DAP-36	*Dermacentor andersoni*	T cells		Bergman et al. (1995)
IRS-2	*Ixodes ricinus*	Neutrophils	Inhibits cathepsin G and chymase	Chmelar et al. (2011)
IRAC I and II	*I. ricinus*	Complement	Dissociates C3 convertase	Schroeder et al. (2007)
IRIS	*I. ricinus*	Monocytes, macrophages, T cells	↓ TNF-α and IFN-γ	Prevot et al. (2009), Fontaine et al. (2011)
Ir-LBP	I. ricinus	Neutrophils (chemotaxis)	Binds leukotriene B4	Beaufays et al. (2008)
BIP	*I. ricinus*	B cells	Inhibits B cell activation	Hannier et al. (2004)
TSLPI	*Ixodes *spp*.*	Complement, neutrophils	Inhibits mannose-binding lectin	Schuijt et al. (2011)
Salp15	*Ixodes *spp.	Dendritic cells, T cells, complement	Raf-1/ MEK activation ↓ IL-6, TNF-α, and IL-12p35 CD4 binding ↓ T cell activation and IL-2 ↓ Membrane attack complex	Hovius et al. (2008), Anguita et al. (2002), Garg et al. (2006), Berende et al. (2010)
Histamine binding proteins (HBP) Lipocalins	*Ixodes* spp. *Rhodnius prolixus*	Basophils, mast cells	Binds histamine	Paesen et al. (1999), Sangamnatdej et al. (2002), Andersen et al. (2005)
Nitrophorins	*Rh. prolixus*		Binds histamine	Gazos-Lopes et al. (2012)
IgG-BP	*Ixodes spp. Rhipicephalus appendiculatus*	IgG	Binds IgG	Wang and Nuttall (1995), Wang and Nuttall (1999), Wang et al. (1998)
Maxadilan	*Lutzomyia longipalpis*	T cells, macrophages	↓ Nitric oxide, TNF- α ↑ Prostaglandin E2, IL-10, IL-6	Gillespie et al. (2000)
Adenosine and adenosine monophosphate	*Phlebotomus papatasi Rhipicephalus sanguineus*	T cells, macrophages, NK cells?, dendritic cells	↓ Nitric oxide and IFN-γ	Hall and Titus (1995), Katz et al. (2000), Oliveira et al. (2011)
Evasin-1, -3, -4	*R. sanguineus Tick* spp.		Binds chemokines (Evasin-1: CCL3, CCL4, CCL18) (Evasin-3: CXCL8, CXCL1) (Evasin-4: CCL5, CCL11)	Frauenschuh et al. (2007), Déruaz et al. (2008)
Ado	*R. sanguineus*	Dendritic cells	Induce cAMP-PKA to reduce cytokine production	Oliveira et al. (2011)
D7 proteins	*Aedes aegypti Anopheles gambiae*		Binds histamine	Calvo et al. (2006)
Sialokinins	*Aedes aegypti*	T cell		Zeidner et al. (1999)

An example of an immunomodulatory molecule in saliva is evasin. This protein manipulates immune signaling by binding key chemokines, thus, inhibiting the production of cytokines (Frauenschuh et al., 2007; Déruaz et al., 2008). The tick proteins ISL929, ISL1373, sialostatin L, IRS-2, Ir-LBP, and TSLP1 all target neutrophils, usually the first immune cell to respond to a pathogen (Kotsyfakis et al., 2006,^2007^; Beaufays et al., 2008; Guo et al., 2009; Sá-Nunes et al., 2009; Chmelar et al., 2011; Schuijt et al., 2011). Antigen presenting cells are the focus of the following salivary molecules: japanin, sialostatin L, PGE_2_, IRIS, Salp15, Ado, and maxadilan (Gillespie et al., 2000; Anguita et al., 2002; Leboulle et al., 2002; Garg et al., 2006; Kotsyfakis et al., 2006,^2007^; Hovius et al., 2008; Schuijt et al., 2008; Prevot et al., 2009; Sá-Nunes et al., 2007,^2009^; Berende et al., 2010; Fontaine et al., 2011; Oliveira et al., 2011; Preston et al., 2013; Ullmann et al., 2013). Histamine release factor (HRF) and histamine binding proteins (HBP) both act on granule releasing cells (Paesen et al., 1999; Sangamnatdej et al., 2002; Mulenga et al., 2003; Andersen et al., 2005; Dai et al., 2010), while sialostatin L affects cytokine secretion by mast cells (Horka et al., 2012). The complement cascade is a crucial factor involved in directing inflammatory responses through the formation of complexes on the pathogen surface, opsonization, and membrane-attack complex (MAC). ISAC, Salp20, IRAC I/II, TSLP1, and Salp15 can all inhibit the complement system (Valenzuela et al., 2000; Anguita et al., 2002; Garg et al., 2006; Tyson et al., 2007; Schroeder et al., 2007; Schuijt et al., 2008; Hovius et al., 2008; Berende et al., 2010). Salivary constituents not only aim for the innate immune system, but they also act on the adaptive immunity. Salivary components may act on T cells, B cells, or antibodies, as is the case with IL-2 binding protein, IsSMase, IRIS, BIP, Salp15, IgG-BP, and maxadilan (Wang and Nuttall, 1995,^1999^; Wang et al., 1998; Gillespie et al., 2001; Hannier et al., 2004; Alarcon-Chaidez et al., 2009; Prevot et al., 2009; Fontaine et al., 2011).

Although some of these proteins have overlapping cellular targets, their activity at the molecular level demonstrate some variability. For instance, ISAC, Salp20, IRAC I/II, TSLP1, and Salp15 inhibit complement through different mechanisms. ISAC, Salp20, and IRACI/II dissociate the crucial complement convertase molecule C3 (Paesen et al., 1999; Lögdberg and Wester, 2000; Valenzuela et al., 2000; Anguita et al., 2002; Leboulle et al., 2002; Sangamnatdej et al., 2002; Andersen et al., 2005; Garg et al., 2006; Daix et al., 2007; Schroeder et al., 2007; Tyson et al., 2007,^2008^; Déruaz et al., 2008; Schuijt et al., 2011). However, TSLP1 and Salp15 target the complement pathway by inhibiting mannose-binding lectin and MAC, respectively (Schuijt et al., 2008). Even within the same organism, salivary proteins can influence T cells in different ways. IL-2 binding, does as its name implies, blocks IL-2 while IsSMase affects T cells by increasing IL-4 (Gillespie et al., 2001; Alarcon-Chaidez et al., 2009). Immune regulation by the saliva of arthropod vectors discussed in this review consists of: (1) impediment of attachment, (2) reduction of oxidants, (3) decrease of pro-inflammatory enzymatic activity, (4) modification of cytokine levels, (5) attenuation of co-receptor binding, and (6) sequestration of pro-inflammatory mediators from binding to their receptors (**Table 2**).

Modulation of host immunity favors arthropod blood-feeding (Fontaine et al., 2011). This occurrence was first observed upon infection with *Leishmania* parasites (Titus and Ribeiro, 1988). More recently, studies demonstrated that enhancement of pathogen infection by saliva seems universal (Francischetti et al., 2009; Fontaine et al., 2011). Increased infectivity in the presence of arthropod saliva has been shown for pathogens transmitted by sandflies, mosquitoes and ticks (Titus et al., 2006). Specifically, mosquito saliva enhances transmission of malaria parasites (Vaughan et al., 1999), West Nile (Styer et al., 2011), La Crosse (Osorio et al., 1996) and Cache Valley (Edwards et al., 1998) viruses. Similarly, tick saliva counteracts host-derived inflammation (Francischetti et al., 2009; Fontaine et al., 2011) by impairing the function of innate and adaptive immune cells (de Silva et al., 2009), and inhibiting cytokine secretion (Fontaine et al., 2011). *Borrelia burgdorferi* – the Lyme disease agent - appears shielded by a salivary protein called Salp15 from the tick *I. scapularis*, and in turn, protected from antibody-mediated killing (Ramamoorthi et al., 2005) and dendritic cell function (Valenzuela et al., 2000; Hovius et al., 2008). However, this effect is not unique to Salp15 because sialostatin L2, another protein, also facilitates pathogen transmission at the skin site (Kotsyfakis et al., 2010). Interestingly, in the *Aedes aegypti* mosquito model, saliva appears to protect dendritic cells from infection with dengue virus *in vitro *(Ader et al., 2004).

An intriguing aspect of the pathogen-saliva interaction lies in the response of the skin to infection (Frischknecht, 2007; Krause et al., 2009). During the infectious blood meal, the arthropod mouthpart dilacerates and penetrates the epidermis and reaches the dermis. The skin injury leads to a local inflammatory response involving secretion of chemokines, cytokines, and antimicrobial molecules as well as dermal mast cell degranulation, fluid extravasation and neutrophil influx (Boulanger et al., 2006; Rubin and Strayer, 2012). This response has a major impact on furthering the establishment of infection because pathogen inoculation follows an arthropod bite. Cellular responses promoted by mast cells, neutrophils, dendritic cells and infiltrated macrophages aim not only to repair the skin injury, but also remove a microbial threat during vector transmission. This series of steps also reverberates on the later activation of adaptive immunity and recruitment of cell types that may promote pathogen propagation in the host, especially for intracellular microorganisms.

## NOD-LIKE RECEPTORS

Approximately two decades ago, a group of sensors were added to the pattern recognition receptor family, expanding what was known about intracellular recognition of endogenous and exogenous molecules (Inohara et al., 1999). NLRs are appropriately named due to their characteristic nucleotide binding and oligomerization domain (NOD). NLRs may also contain leucine-rich repeats (LRR) at their C-terminus and a variable effector domain at their N-terminal end, all of which play a role in pathogen recognition and immunity (Moreira and Zamboni, 2012). Although 22 human and 30 mouse NLRs been discovered, to stay within the scope of our review, we will only address those that have been associated with crucial vector-borne diseases (**Table 1**; Schroder and Tschopp, 2010; Moreira and Zamboni, 2012).

## NOD1 AND NOD2

**FIGURE 1:**
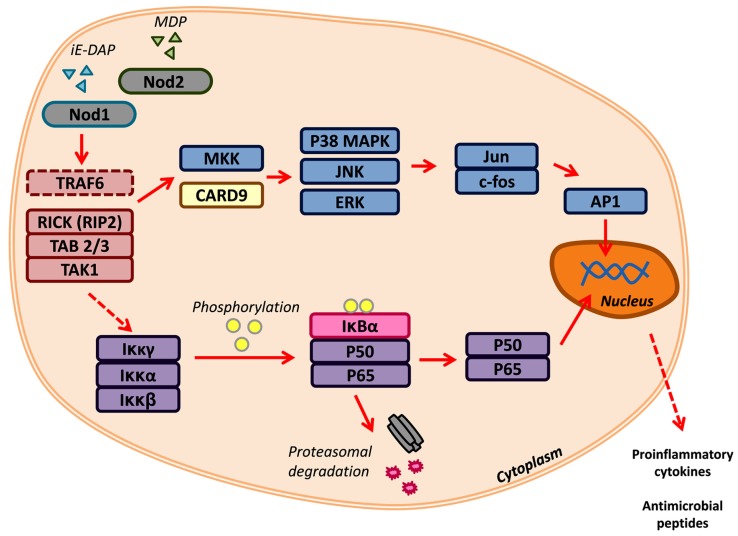
**Nod1 and Nod2 signaling.** Nod1 and Nod2 are activated by the peptidoglycan components iE-DAP and MDP, respectively. Recognition of PAMPs triggers TRAF6, RICK/RIP2, TAB 2/3, and TAK1. These can signal downstream to two major signaling networks: (1) MAP kinase and the (2) NF-κB pathways. Transcription factors, such as AP1 and the NF-κB complex (p50/p65), translocate to the nucleus to promote the transcription of pro-inflammatory cytokines and antimicrobial peptides.

Nod1 and Nod2 are crucial for the recognition of peptidoglycan components (**Figure 1**). Signaling through Nod1 and Nod2 begins with the initiation of Nod1 by D-glutamyl-meso-diaminopimelic acid (DAP) and/or Nod2 by muramyl dipeptide (MDP; Chamaillard et al., 2003; Girardin et al., 2003a). While the NOD portion acts as a receiver in the presence of these pathogenic molecules, the effector CARD domain(s) of Nod1 and Nod2 perpetuate the signal transduction by interacting with receptor-interacting serine/threonine protein kinase-2 (RIP2/RICK; Kobayashi et al., 2002). Classically, RIP2/RICK is polyubiquitinated by TNF receptor-associated factor 6 (TRAF6), this signal is required for the recruitment of the adaptor molecules TAK1-binding protein 2 and 3 (TAB2/3) and activation of TAK1 (Besse et al., 2007). Together this forms the TGF-β-activated kinase 1 (TAK1) complex that promotes the degradation of the inhibitor of nuclear factor (NF)-κB, thereby allowing the translocation of NF-κB into the nucleus. This is only one signaling cascade that is activated by Nod1/2, the mitogen-activated protein kinases (MAPK) pathway is another branch that can be driven by these NLRs (Pauleau and Murray, 2003; Park et al., 2007). Nod1 and Nod2 can activate three key MAPK: extracellular signal-related kinases (ERK), Jun amino-terminal kinases (JNK), and p38. The latter two can also be signaled by Nod2 through the adaptor caspase recruitment domain-containing protein 9 (CARD9; Colonna, 2007). The activation of each pathway results in the expression of pro-inflammatory mediators, such as cytokines and antimicrobial peptides. Nod1 and Nod2 can be regulated by A20-mediated ubiquitin modifications and caspase-12 inhibition of RIPK2-TRAF6 complex formation (Hitotsumatsu et al., 2008; LeBlanc et al., 2008).

Recent developments have identified a new role for Nod1 and Nod2 in the recognition of pathogens lacking peptidoglycan. Studies have reported that Nod proteins can respond to protozoan parasites, like *Toxoplasma gondii* (Shaw et al., 2009). Surprisingly, Nod2 has been shown to respond to single-stranded RNA (Sabbah et al., 2009). The activation of Nod2 in this case is dependent upon the mitochondrial antiviral signaling protein MAVS and results in the facilitation of interferon regulatory factor 3 (IRF3) mediated interferon (IFN) gene expression. Another protective measure that Nod1 and Nod2 are involved in is the induction of autophagy related 16-Like 1 (ATG16L1)-dependent autophagy in response to bacterial invasion, such is the case with *Listeria monocytogenes *(Travassos et al., 2010). Nod1 and Nod2 are gradually revealing their complex nature. Most commonly acknowledged as a sensor for peptidoglycan molecules, there is also debate that Nod1 and Nod2 may possess regulatory abilities (Murray, 2005). Studies regarding Nod1 and Nod2 function are continuously being assessed in order to develop a comprehensive understanding of these key proteins.

## INFLAMMASOME

**FIGURE 2:**
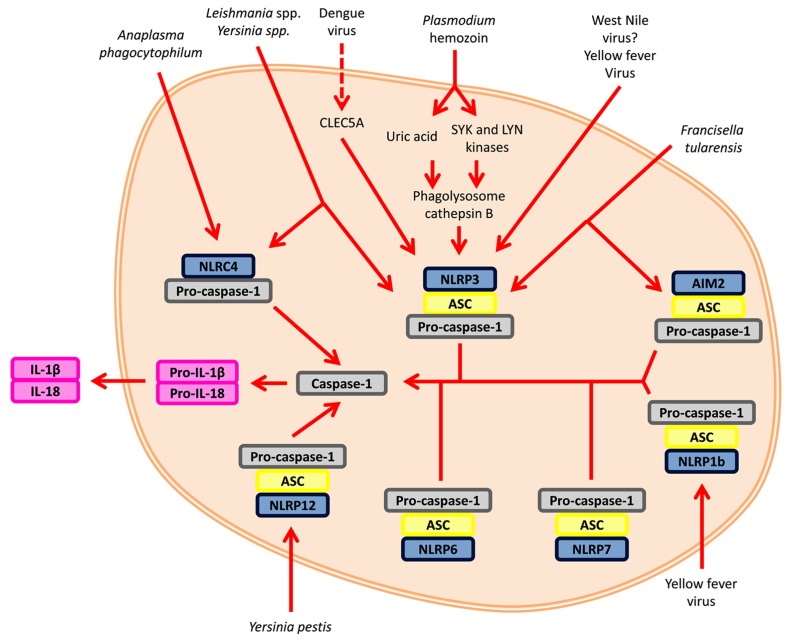
**Inflammasome signaling.** The inflammasome is activated by two signals. The first signal (*not shown*) involves the recognition of an agonist/PAMP by a receptor, such as TLRs, and upregulates transcription of pro-inflammatory cytokines and key NLRs. Signal two is the initiation of the inflammasome itself. Various inflammasomes can be activated by vector-borne pathogens. The activation and oligomerization of the inflammasomes converge upon the activation of caspase-1 and the maturation of pro-inflammatory cytokines, such as IL-1β and IL-18.

The inflammasome is a potent innate immune structure characterized by its ability to activate pro-caspase-1 in response to PAMPs or DAMP (**Figure 2**). The inflammasome scaffold is created by the oligomerization and recruitment of several proteins. One component, the receptor, defines the inflammasome; it can either originate from the NLR family or contain the HIN-200 domain (Lamkanfi and Dixit, 2011). Depending upon the receptor type, the adaptor molecule ASC may or may not be implicated. Since ASC possesses both a pyrin and CARD domain, it facilitates the association between the CARD-containing pro-caspase-1 and a receptor lacking the CARD domain (Schroder and Tschopp, 2010). Classically, inflammasome-mediated cytokine secretion is the product of a two-tiered signaling system (**Figure 2**; Franchi et al., 2012). The first signal concerns the activation the NF-κB pathway in order to promote the gene expression of IL-1β and IL-18 and other pro-inflammatory genes, such as *Nlrp3*. The second signal involves the assembly of the inflammasome, which results in the secretion of the abovementioned cytokines. Common to all canonical inflammasomes is the presence of the enzyme pro-caspase-1. Caspase-1 is responsible for the maturation of the pro-inflammatory cytokines interleukin (IL)-1β and IL-18 and the inflammation-related cell death process termed pyroptosis (Davis et al., 2011). Other caspases have also been shown to be involved in the inflammasome signaling pathway. Caspase-11 was recently discovered to modulate caspase-1 in response to certain Gram-negative bacteria, such as *Citrobacter rodentium *(Kayagaki et al., 2011; Rathinam et al., 2012). Another non-canonical inflammasome involves caspase-8. Caspase-8 is a negative regulator of pro-inflammatory NLRP3 inflammasome activity (Kang et al., 2013). During macrophage infection with *Francisella tularensis* subspecies *novicida*, caspase-8 can form a complex with AIM2 and ASC (Pierini et al., 2012). Caspase-8 associates with dectin-1 in the presence of fungi and mycobacteria (Gringhuis et al., 2012). Caspase-5 can also bind with an inflammasome, namely NLRP1 (Martinon et al., 2002). Not only can caspases bind to the inflammasome, they can also be cleaved by the caspase-1 component of the protein scaffold, similar to IL-1β. This phenomenon is seen in caspase-7 activation by caspase-1 during *Legionella pneumophila *infection (Akhter et al., 2009). Taken together, multiple checkpoints are crucial for inflammasome regulation due to its strength as a pro-inflammatory initiator.

## RECOGNITION OF VECTOR-BORNE PATHOGENS BY NLRs

Medically relevant vector-borne pathogens have plagued the health of individuals all over the globe (**Table 1**). Even more concerning is the rate at which these diseases are escalating and claiming the lives of thousands of people (Hotez et al., 2009). The relationship between these daunting pathogens and recognition by NLRs is not fully understood.

## NOD1 AND NOD2

Being one of the first NLRs discovered, many studies have been aimed to the role of Nod1 in the context of bacterial pathogenesis (Chamaillard et al., 2003; Girardin et al., 2003b; Ray et al., 2009). Research involving the sensing of bacteria in the intracellular compartment of a wide range of cell types has dominated the Nod1 field. However, Silva et al. (2010) were able to classify Nod1 as a crucial component for the resistance to the parasite *Trypanosoma cruzi*. *T. cruzi* is transmitted by the kissing bug, *Rhodnius prolixus*, primarily in Latin American countries. It is the causative agent of Chagas disease, which can be characterized by fever, edema, or inflammation in the heart and/or brain (Centers for Disease Control and Prevention, 2010). These authors observed, through the use of Nod1^-^^/^^-^ and Nod2^-^^/^^-^ mice, that IL-12 and TNF-α levels were reduced after infection. Since nitric oxide is a key factor for *T. cruzi* containment, interferon gamma (IFN-γ) was used to treat Nod1^-^^/^^-^ and Nod2^-^^/^^-^ bone marrow-derived macrophages. This resulted in a high load of parasites for the Nod1^-^^/^^-^ macrophage, highlighting the specificity of Nod1, not Nod2, for *T. cruzi* infection.

*B. burgdorferi* is a spirochete transmitted by *Ixodes* spp. Infection by* B. burgdorferi* causes Lyme disease, the most common vector-borne disease north of the equator (Parola and Raoult, 2001; Lindgren and Jaenson, 2006; Berende et al., 2010). Lyme disease can manifest into a three stage infection: (1) erythema migrans is characterized by localized infection, (2) early disseminated infection results in inflamed joints and CNS, and (3) persistent infection, which consists of chronic inflammation of joints and the CNS and sensory polyneuropathy (Berende et al., 2010). It has been established that TLR2 plays an important role in the recognition of *B. burgdorferi*. Recent evidence points to Nod2 as an important factor in the sensing of this pathogenic spirochete (Petnicki-Ocwieja et al., 2011). Nod2 is upregulated in mouse microglia and individuals with mutated Nod2 were not able to mount an efficient cytokine response after infection with *B. burgdorferi* (Sterka et al., 2006; Oosting et al., 2010). The plague causing vector-borne pathogen *Yersinia* has also been shown to be recognized by Nod2 (Ferwerda et al., 2009).

Nod1 and Nod2 also appear to possess redundancy because they are able to detect similar arthropod-borne pathogens. Individuals who encountered an antigenic component from the *Brugia malayi* adult demonstrated an increase in Nod1 and Nod2 expression (Babu et al., 2009). *Brugia* and *Wuchereria bancrofti *species can cause lymphatic filariasis which can manifest as elephantiasis, lymphedema, and hydrocele (Centers for Disease Control and Prevention, 2013a). Independently, the obligate intracellular pathogen *Anaplasma phagocytophilum*, transmitted by *Ixodes* spp., is involved in the increased expression of Rip2, a critical molecule in Nod1 and Nod2 signaling (Sukumaran et al., 2012). More importantly, the ability for *Rip2*^-^^/^^-^ mice to control and clear *A. phagocytophilum* was severely hindered. The *Plasmodium* parasite is also detected by Nod proteins (Coban et al., 2007). Certain instances result in upregulation of Nod2 in the presence of *Plasmodium* sporozoites, while in other cases Nod1 and Nod2 confer changes in cytokines but do not promote survival after infection (Ockenhouse et al., 2006; Finney et al., 2009).

## NLRP1 INFLAMMASOME

The NLRP1 inflammasome was the first to be characterized (Martinon et al., 2002). NLRP1 has been shown to recognize the *Bacillus anthracis* lethal toxin and, like Nod2, MDP (Boyden and Dietrich, 2006; Faustin et al., 2007). The activation of pro-caspase-1 activity elicited by these bacterial components is distinct. Cleavage of the NLRP1 inflammasome by the lethal toxin is required for inflammasome activation, as mutation of NLRP1 demonstrates reduced caspase-1 activation (Levinsohn et al., 2012). On the other hand, MDP activation of NLRP1 requires the presence of MDP and ribonucleoside triphosphates (Faustin et al., 2007). It was observed that a cohort given a yellow fever vaccine showed upregulation of caspase-1 and caspase-5. These two caspases are present in the NLRP1 inflammasome. This indicates that the NLRP1 inflammasome may be activated by the yellow fever virus. This virus is transmitted by the mosquito *Aedes aegypti*. Inoculation of yellow fever virus by a mosquito can lead to mild reactions, such as fever, ache, and nausea, or more serious ones, such as organ failure (Centers for Disease Control and Prevention, 2011a). More studies need to be done in order to clarify what components trigger a NLRP1 inflammasome response to the yellow fever virus.

## NLRP3 INFLAMMASOME

Of all NLRs, NLRP3, currently, has the most known associations with vector-borne diseases. It is well known that NLRP3 is triggered by three signals: (1) potassium efflux, (2) phagolysosomal disruption, and (3) ROS production (Schroder and Tschopp, 2010). Recently, mitochondrial DNA and calcium levels were suggested to be other activators of the NLRP3 inflammasome (Rossol et al., 2012; Shimada et al., 2012). The malarial parasite has demonstrated the ability to activate the NLRP3 inflammasome through the crystalline particle hemozoin (Dostert et al., 2009; Griffith et al., 2009; Shio et al., 2009). Monosodium urate (uric acid), together with hemozoin, has also been reported to result in pro-inflammatory reactions through the MAPK signaling pathway (Griffith et al., 2009; Shio et al., 2009). Hemozoin is a byproduct of heme detoxification by *Plasmodium*. The phagocytosis of hemozoin initiates signals through spleen tyrosine kinase (Syk) and v-yes-1 Yamaguchi sarcoma viral related oncogene homolog (Lyn), tyrosine kinases, in order to initiate the NLRP3 inflammasome (Shio et al., 2009). Another mosquito-borne pathogen, the dengue virus is transmitted by *A. aegypti* or *A. albopictus*. Dengue virus can cause dengue fever or dengue shock syndrome. Wu et al. (2013) elucidated that, in human macrophages, dengue virus can signal through Syk-coupled C-type lectin 5A (CLEC5A) to induce NLRP3-mediated cytokine secretion and pyroptosis. Though not much is known about yellow fever virus and the inflammasome, one study shows that vaccination with a live attenuated yellow fever vaccine is able to increase the expression caspase-1 associated with the NLRP3 inflammasome (Gaucher et al., 2008).

IL-1β is crucial for the protection of the CNS from West Nile neuroinvasive disease (Ramos et al., 2012). Moreover, it was shown that this phenomenon is specific for NLRP3 inflammasome mediated IL-1β secretion. Additionally, IL-1β combined with type I IFN results in the reduction of West Nile virus infection. Non-mosquito-borne pathogens also influence NLRP3 activity. Infection by *Leishmania* spp., transmitted by the sandfly *Lutzomyia longipalpis*, can result in skin, organ, and/or mucosal complications (Centers for Disease Control and Prevention, 2013b). In murine macrophages, Sani et al. (2013) found that the expression of *Nlrp3* is increased after exposure to *Leishmania* *major*. Furthermore, Lima-Junior et al. (2013) confirmed NLRP3 activation after *L. amazonensis* infection that led to the protective restriction of parasites. Another non-mosquito-borne pathogen is *Francisella tularensis*, which is commonly transmitted by ticks. Tularemia can cause sores and respiratory complications. Uniquely in human leukemia cell line (THP-1) but not in mouse cells, *Francisella* is capable of activating the NLRP3 inflammasome (Atianand et al., 2011). Supporting this, the use of NLRP3 inflammasome inhibitors and *Nlrp3* siRNA revealed that the IL-1β secretion in response to *Francisella* was lessened. The type III secretion system (T3SS) from *Yersinia pestis* is also able to activate the NLRP3 inflammasome *in vitro *(Brodsky et al., 2010). With the addition of KCl, the NLRP3 inflammasome activity was nullified. However, other inflammasomes are also involved in the detection of *Yersinia* as well. Although Nod2 has been acknowledged as a protein that recognizes *Borrelia*, there is controversy on whether inflammasomes are activated in response to this vector-borne pathogen. Though independent of the NLRP3 inflammasome, multiple groups have found that caspase-1 is activated after exposure to *Borrelia* while another group was unable to detect caspase-1 dependence (Cruz et al., 2008; Liu et al., 2009; Oosting et al., 2011).

## NLRC4 INFLAMMASOME

The CARD-containing NLRC4 inflammasome mediates pro-inflammatory responses to the recognition of flagellin and type III/IV secretion systems from gram-negative bacteria (Schroder and Tschopp, 2010). NLRC4, also called IPAF, inflammasome confer protection against bacteria, such as *Salmonella typhimurium *and *Pseudomonas aeruginosa *(Miao et al., 2008). It is also able to directly and indirectly associate with pro-caspase-1, via its CARD domain or the adaptor molecule ASC, respectively. Additionally, another level of specificity is added by the NLRC4 interaction with NAIP5 or NAIP2, which modifies NLRC4 activation in response to flagellin and the type III secretion system (T3SS), respectively (Zhao et al., 2011). As of yet, NLRC4 has been implicated in two vector-borne illnesses, Human granulocytic anaplasmosis and Leishmaniasis. *Nlrc4*^-^^/^^-^ mice showed heightened susceptibility to *Anaplasma phagocytophilum* and decreased levels of IL-18 relative to the wild-type. However, the effect of NLRC4 was partial; thereby, suggesting additional mechanisms of inflammasome activation (Pedra et al., 2007). Sani et al. (2013) found that *Nlrc4* expression increased after exposing macrophages to *L. major*. As was previously mentioned, *Y. pestis* is able to activate several inflammasomes, and it is also able to combat this recognition with effector proteins (Brodsky et al., 2010). The NLRC4 inflammasome is another protein complex involved in the recognition of *Y. pestis* T3SS (Brodsky et al., 2010).

## NLRP12 INFLAMMASOME

The NLRP12 inflammasome is a member of the NLR family that has been suggested to reduce and potentiate inflammatory cytokine secretion (Wang et al., 2002; Lich et al., 2008; Arthur et al., 2010; Zaki et al., 2011; Allen et al., 2012). Currently, NLRP12 has been shown to play a role in hereditary period fever syndromes, but very little is known with respect to vector-borne diseases. Vladimer et al. (2012) discovered that NLRP12 regulates IL-18 secretion in response to *Y. pestis*. More specifically, after infection of *Nlrp12*^-^^/^^-^ mice with *Y. pestis*, they observed an increase in bacterial load and death which was associated with decreased levels of IL-18 and IL-1β.

## NON-NLR INFLAMMASOME

The AIM2 (absent in melanoma 2) inflammasome does not contain the typical NLR domain as do other inflammasomes. Rather, it carries the HIN-200 domain (Case, 2011). In particular, AIM2 is known for sensing double stranded DNA in the cytosol (Bauernfeind et al., 2011). The formation of the AIM2 inflammasome consists of the AIM2 receptor, ASC, and pro-caspase-1. Upon recognition of cytoplasmic DNA, AIM2 is able to coordinate pyroptosis and the release of IL-1β and IL-18 via pro-caspase-1 maturation (Davis et al., 2011). Of the vector-borne pathogens discussed in this review, AIM2 is able to recognize *F. tularensis* in mouse macrophages (Fernandes-Alnemri et al., 2010). Moreover, IRF3 is needed for a type 1 IFN response to help mount an effective AIM2-dependent activation after *F. tularensis* infection (Fernandes-Alnemri et al., 2010).

## CONCLUDING REMARKS

The importance of NLRs and vector saliva has been demonstrated through numerous elaborate studies. Further research in this area has the potential to reveal more intricate relationships, as well as the salivary effectors that can modulate these interactions. This review has highlighted the role of NLRs and salivary components in vector-borne diseases. Due to the vast amount of literature available in the field of arthropod saliva and the diverse mechanisms of vertebrate-host immunomodulation, we elected to focus only on those pertinent to the vectors discussed here. Elucidating the mechanisms behind NLR recognition and salivary modulation of pathogenic agents will shed light on the fundamental basis of pathogen-vector-host interactions. Additionally, it should provide novel targets for therapeutic intervention of devastating vector-borne diseases.

Based on our current knowledge, we suggest that arthropod saliva could regulate NLR inflammasome activity during pathogen transmission or after infection. Vector saliva has been shown to minimize reactive oxygen species (ROS; Guo et al., 2009). ROS has been identified as an agonist for inflammasome activation; therefore salivary proteins can potentially reduce ROS to decrease inflammasome activity. Another mechanism by which arthropod saliva can hinder the inflammasome is by acting on caspase-1. Caspase-1, the key enzymatic component of the inflammasome, is a member of the cysteine protease family. Salivary proteins have demonstrated the ability to target cysteine proteases, such as sialostatin L inhibition of cathepsin L (Kotsyfakis et al., 2006). Of interest, the same protein exhibits anti-inflammatory effects. Thus, it is plausible that sialostatins block caspase-1 activation and subsequent IL-1β and IL-18 secretion. A better understanding of salivary components regulating vector-borne pathogens and NLR interaction could allow us to gain a foothold on controlling these infectious diseases.

## AUTHOR CONTRIBUTIONS

Olivia S. Sakhon, Maiara S. Severo, Michail Kotsyfakis, and Joao H. F. Pedra wrote the manuscript. Olivia S. Sakhon created the tables and figures.

## Conflict of Interest Statement

The authors declare that the research was conducted in the absence of any commercial or financial relationships that could be construed as a potential conflict of interest.
